# Supplementary material for: SLC5A1 Variants in Turkish Patients with Congenital Glucose-Galactose Malabsorption
Source: Genes (Basel). 2023 Jun 27;14(7):1359. doi: 10.3390/genes14071359 (PMC10379334; doi:10.3390/genes14071359)
Supplement: Supplementary file 1 [file genes-14-01359-s001.zip › genes-2179955-supplementary.pdf]

Figure S1 Original images overview

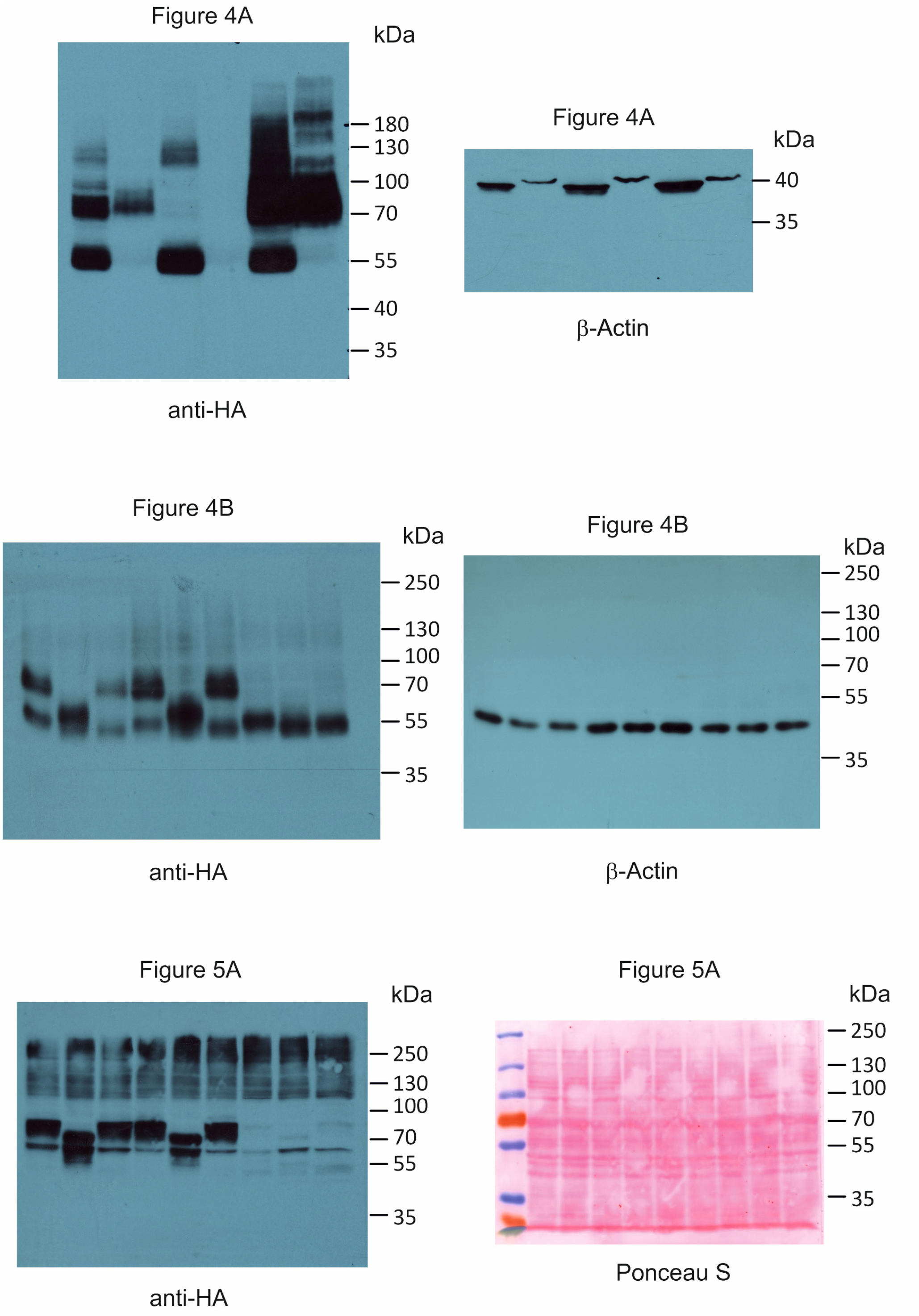

Original gel images refer to the Figures in the main document as indicated by designations "Figure 4A", "Figure 4B" and "Figure 5A".
